# Advances in immunotherapy for gastrointestinal malignancies

**DOI:** 10.3389/fimmu.2026.1969637

**Published:** 2026-10-14

**Authors:** Bo Liu, Lei Wang, Yuehan Ma, Weijie Xu, Xinting Chen, Zhaocheng Chi, Baocheng Xing

**Affiliations:** 1 Second Department of Gastrocolorectal Surgery, Jilin Cancer Hospital, Changchun, China; 2 First Department of Gastrocolorectal Surgery, Jilin Cancer Hospital, Changchun, China; 3 Department of Medical Oncology, Jilin Cancer Hospital, Changchun, China; 4 Department of Endoscopy, Jilin Cancer Hospital, Changchun, China

**Keywords:** biomarkers, cellular therapy, gastrointestinal malignancies, immune checkpoint inhibitors, perioperative immunotherapy, treatment toxicity

## Abstract

Gastrointestinal malignancies remain leading causes of cancer mortality, yet their sensitivity to immunotherapy differs sharply across anatomical sites, histologies, molecular subtypes, and disease stages. Immune checkpoint blockade has established durable clinical benefit in microsatellite instability-high colorectal cancer, advanced gastric and esophageal cancers, hepatocellular carcinoma, and biliary tract cancer, while pancreatic ductal adenocarcinoma and microsatellite-stable colorectal cancer remain largely refractory. Recent phase III trials have moved immunotherapy into perioperative treatment. Concurrently, claudin 18.2-directed chimeric antigen receptor T cells, T-cell receptor-engineered cells, bispecific antibodies, personalized vaccines, and oncolytic viruses are converting previously speculative strategies into clinically testable platforms. This review integrates contemporary trial evidence with mechanisms of immune escape, biomarker development, and combination toxicity. The resulting framework prioritizes evidence maturity across diverse gastrointestinal tumor types worldwide.

## 1 Introduction

Gastrointestinal malignancies arise in the esophagus, stomach, colorectum, liver, biliary tract, and pancreas. GLOBOCAN 2022 estimated nearly 20 million new cancer cases and 9.7 million deaths worldwide; gastrointestinal malignancies remain prominent contributors to this burden (1). Their immunobiology, however, is not interchangeable across disease sites. Viral antigens, mismatch-repair deficiency, tumor mutational burden, stromal architecture, chronic inflammation, and organ-specific immune tolerance create distinct therapeutic contexts. The tumor-agnostic activity of programmed cell death protein 1 (PD-1) blockade in mismatch repair-deficient tumors established genomic instability as a clinically actionable immune phenotype, but subsequent experience showed that no single biomarker captures the full probability of response (2, 3). Immune checkpoint inhibitors now anchor treatment in several advanced gastrointestinal cancers and increasingly enter neoadjuvant, perioperative, and adjuvant settings. At the same time, negative trials and modest absolute benefits caution against describing immunotherapy as uniformly transformative. HLA loss, defective antigen presentation, T-cell exclusion, and therapy-induced immune editing can uncouple neoantigen load from effective tumor rejection (4, 5). This review summarizes the biological basis and clinical progress of immunotherapy across gastrointestinal cancers and discusses emerging biomarkers, combination strategies, and approaches to overcome therapeutic resistance.

## 2 Immunotherapy in gastrointestinal malignancies

### 2.1 Immune ecosystems and tumor immunogenicity

The gastrointestinal immune system must tolerate food antigens and commensal organisms while retaining antimicrobial and antitumor competence; cancers exploit this regulatory architecture through tissue-specific combinations of myeloid suppression, regulatory T cells, dysfunctional cytotoxic lymphocytes, and cancer-associated fibroblasts. In pancreatic ductal adenocarcinoma (PDAC), dense extracellular matrix, hypovascularity, granulocytic and monocytic suppressor populations, and limited dendritic-cell function restrict both drug delivery and T-cell priming. Colorectal cancer illustrates the opposite extreme: mismatch repair-deficient (dMMR) tumors can contain abundant mutation-derived neoantigens and activated lymphocytes, whereas most mismatch repair-proficient tumors remain immune excluded or immune indifferent (2, 6). Single-cell analysis further shows that response cannot be reduced to immune-cell abundance; differentiation state, spatial location, antigen specificity, and interactions with stromal cells determine whether infiltrating lymphocytes remain functional (7). Tumor mutational burden (TMB) increases the probability of generating immunogenic peptides but is neither a direct measure of antigen quality nor a universal predictor. Effective rejection additionally requires intact proteasomal processing, transporter-associated antigen presentation, HLA expression, dendritic-cell priming, trafficking, and sustained effector function. Allele-specific HLA loss and beta-2-microglobulin disruption can remove immunogenic clones from immune recognition, while JAK1/2 pathway alterations may impair interferon responsiveness despite ongoing T-cell pressure (4, 8). In metastatic colorectal cancer models, transforming growth factor beta (TGF-β) produced by stromal compartments excludes T cells and limits checkpoint-blockade activity, providing a mechanistic rationale for combinations that alter the microenvironment rather than simply adding another inhibitory-receptor antibody (5). These observations support composite immune phenotyping over reliance on a single genomic threshold.

### 2.2 Checkpoint regulation and determinants of response

PD-1 ligation attenuates proximal T-cell receptor signaling and stabilizes dysfunctional states during chronic antigen exposure, whereas cytotoxic T-lymphocyte-associated protein 4 (CTLA-4) constrains costimulation during priming and contributes to regulatory T-cell function. Blocking these pathways can expand pre-existing tumor-reactive clones, recruit new clonotypes, and remodel myeloid and stromal compartments, but the magnitude of each effect depends on baseline immune competence. In dMMR colorectal cancer, paired tissue profiling before and after PD-1 blockade demonstrated coordinated remodeling of immune and stromal populations rather than a uniform increase in all T-cell subsets (9). Thus, response should be interpreted as an ecosystem transition rather than a binary release of one inhibitory signal. Programmed death-ligand 1 (PD-L1) scoring has practical value but varies by antibody clone, platform, tumor-cell score, combined positive score (CPS), and tumor area positivity. KEYNOTE-062 showed that first-line pembrolizumab activity in gastric or gastroesophageal junction (G/GEJ) adenocarcinoma was enriched by PD-L1 expression, yet PD-L1 did not cleanly separate all beneficiaries from nonbeneficiaries (10). MSI/dMMR remains the most reproducible tissue-agnostic biomarker, and KEYNOTE-158 extended pembrolizumab activity to noncolorectal MSI-high/dMMR cancers (11). TMB can add information in selected settings, but its cutoff, sequencing platform, tumor-type calibration, and imperfect overlap with effective neoantigens restrict unqualified extrapolation (3) (**Figure 1**).

**Figure 1:**
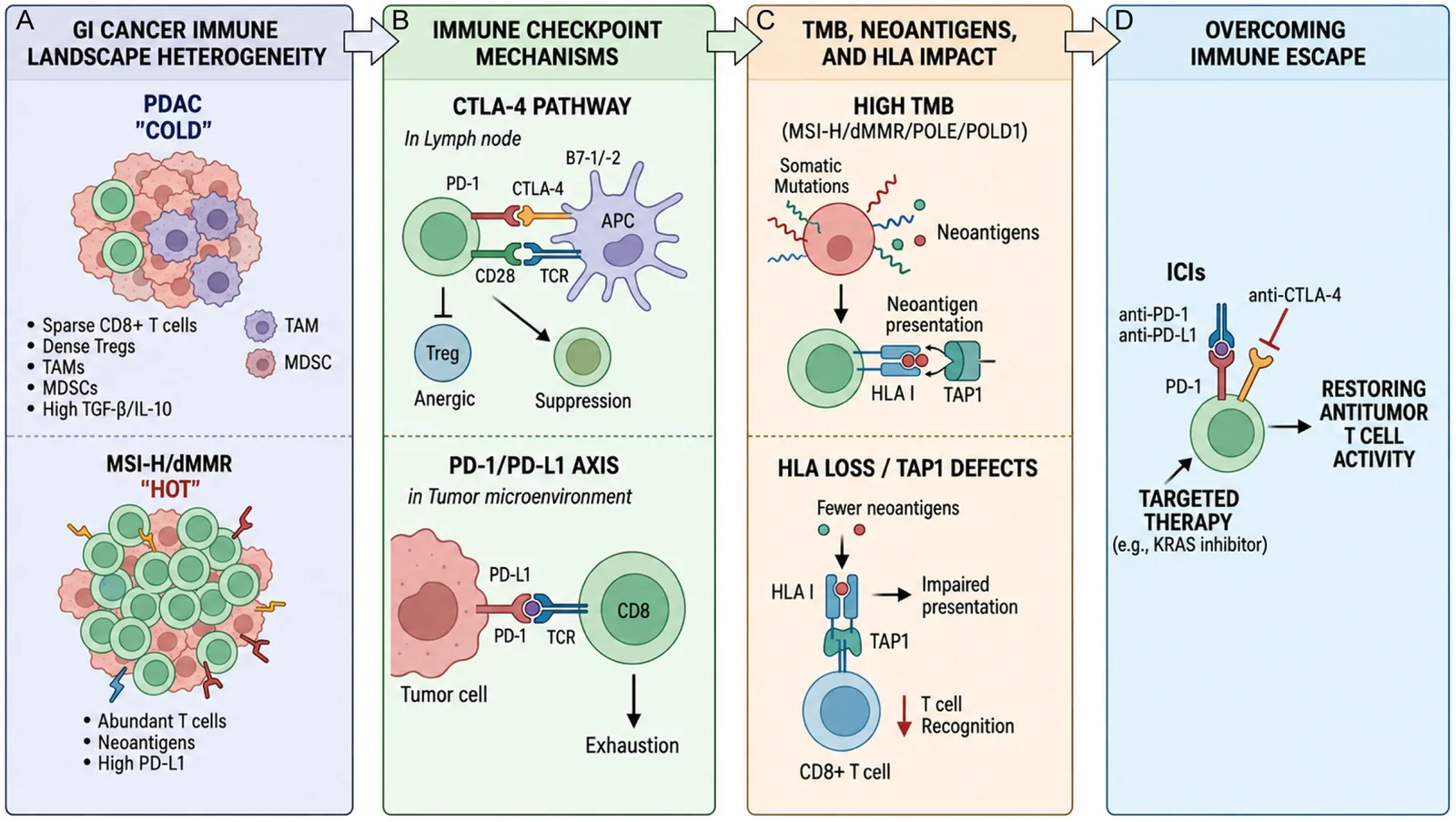
Tumor immune microenvironment in gastrointestinal malignancies. **(A)** Contrasting immune landscapes across gastrointestinal malignancies. Pancreatic ductal adenocarcinoma typically exhibits an immune-cold microenvironment characterized by sparse CD8^+^ T-cell infiltration and enrichment of regulatory T cells, tumor-associated macrophages, myeloid-derived suppressor cells, transforming growth factor-β, and interleukin-10. By contrast, microsatellite instability-high or mismatch repair-deficient tumors frequently display abundant tumor-infiltrating lymphocytes, increased neoantigen burden, and elevated programmed death-ligand 1 expression. **(B)** Principal immune-checkpoint mechanisms. In lymph nodes, cytotoxic T-lymphocyte-associated protein 4 competes with CD28 for B7-1/B7–2 binding, thereby limiting T-cell priming and promoting anergy or regulatory T-cell-mediated suppression. Within the tumor microenvironment, programmed cell death protein 1 engagement by programmed death-ligand 1 attenuates T-cell receptor signaling and promotes CD8^+^ T-cell exhaustion. **(C)** Influence of tumor mutational burden, neoantigens, and antigen-presentation integrity. High tumor mutational burden associated with microsatellite instability, mismatch repair deficiency, or POLE/POLD1 alterations can increase neoantigen generation and HLA class I-mediated presentation, whereas HLA loss or TAP1 dysfunction impairs antigen presentation and reduces cytotoxic T-cell recognition. **(D)** Therapeutic strategies for overcoming immune escape. Immune-checkpoint inhibitors targeting programmed cell death protein 1, programmed death-ligand 1, or cytotoxic T-lymphocyte-associated protein 4, together with genotype-directed therapies such as KRAS inhibitors, may restore or enhance antitumor T-cell activity.

## 3 Clinical advances in immunotherapy for gastrointestinal malignancies

### 3.1 Immune checkpoint inhibitors in gastric and esophageal cancers

First-line immunochemotherapy is established for HER2-negative advanced G/GEJ adenocarcinoma, but the magnitude of benefit varies with PD-L1 enrichment and geography. CheckMate 649 demonstrated longer overall survival with nivolumab plus chemotherapy, with the clearest effect in patients with PD-L1 CPS at least 5; three-year follow-up confirmed durable separation of survival curves (12, 13). KEYNOTE-859 similarly supported pembrolizumab plus chemotherapy, while its China subgroup analysis reinforced applicability to Chinese practice (14, 15). ATTRACTION-4 improved progression-free survival but not overall survival in an Asian population, emphasizing that subsequent therapy, population composition, and biomarker distribution can alter trial interpretation (16, 17). For HER2-positive disease, KEYNOTE-811 established that adding pembrolizumab to trastuzumab and chemotherapy increases response and prolongs progression-free survival, integrating immune and HER2-directed treatment (18).

ORIENT-16 showed that sintilimab plus chemotherapy prolonged overall survival in unresectable G/GEJ cancer, and RATIONALE-305 demonstrated an overall-survival advantage for tislelizumab plus chemotherapy, with continued benefit on longer follow-up (19–21). Cadonilimab, a tetravalent PD-1/CTLA-4 bispecific antibody, improved survival with chemotherapy in the phase III COMPASSION-15 study, including activity in PD-L1-low disease, although grade 3 or higher treatment-related adverse events were more frequent than with chemotherapy alone (22). Earlier COMPASSION-04 data supported the regimen’s clinical development but do not replace the randomized evidence (23). These trials are particularly relevant to Chinese readers because access, approved indications, biomarker assays, and treatment costs differ from Western settings. Perioperative therapy requires separate appraisal because pathological response does not automatically predict event-free or overall survival. KEYNOTE-585 increased pathological complete response, but interim and final analyses did not establish a sufficiently persuasive event-free survival advantage across the overall population to define pembrolizumab plus perioperative chemotherapy as a universal standard (24, 25). By contrast, MATTERHORN showed that perioperative durvalumab plus FLOT improved event-free survival and pathological complete response in resectable gastric or GEJ adenocarcinoma, moving immunotherapy into a curative-intent setting (26) (**Table 1**).

**Table 1:** Representative clinical advances in immunotherapy for gastrointestinal malignancies.

Setting	Representative studies	Intervention	Interpretation
Advanced G/GEJ adenocarcinoma	CheckMate 649; KEYNOTE-859; ORIENT-16; RATIONALE-305	PD-1 inhibitor plus chemotherapy	Established first-line immunochemotherapy; magnitude of benefit varies with PD-L1 assay and population.
Perioperative G/GEJ adenocarcinoma	KEYNOTE-585; MATTERHORN	Checkpoint inhibitor plus perioperative chemotherapy	MATTERHORN improved event-free survival; KEYNOTE-585 did not establish a universal perioperative benefit.
Advanced ESCC	KEYNOTE-590; CheckMate 648; ORIENT-15; RATIONALE-306	PD-1-based first-line regimens	Multiple global and Chinese phase III trials support histology- and biomarker-aware treatment selection.
Resected esophageal/GEJ cancer	CheckMate 577	Adjuvant nivolumab	Standard option after neoadjuvant chemoradiotherapy, R0 resection, and residual pathological disease.
MSI-high/dMMR colorectal cancer	KEYNOTE-177; CheckMate 8HW; NICHE-2	PD-1 blockade with or without CTLA-4 blockade	Supports metastatic first-line therapy and highly active neoadjuvant treatment in localized disease.
Unresectable HCC	IMbrave150; HIMALAYA; ORIENT-32; CARES-310	Checkpoint blockade plus VEGF inhibition or CTLA-4 blockade	Several first-line standards with different bleeding, hepatic, vascular, and immune-toxicity considerations.
Advanced biliary tract cancer	TOPAZ-1; KEYNOTE-966	PD-L1 or PD-1 blockade plus gemcitabine-cisplatin	Validated modest but durable survival improvement over chemotherapy alone.
CLDN18.2-positive gastric/GEJ cancer	CT041 phase I; CT041-ST-01 phase II; IBI389 phase	CAR-T or CLDN18.2xCD3 bispecific antibody	Clinically active next-generation platforms; require specialized toxicity and manufacturing infrastructure.

### 3.2 Esophageal cancer

Checkpoint blockade has changed both metastatic and postoperative management of esophageal cancer. KEYNOTE-590 established pembrolizumab plus chemotherapy as first-line therapy for advanced esophageal cancer, whereas CheckMate 648 showed that nivolumab plus chemotherapy or nivolumab plus ipilimumab improves survival in esophageal squamous cell carcinoma (ESCC), with benefit concentrated among patients with PD-L1-positive tumors for some comparisons (27, 28). These data demonstrate that chemotherapy-free dual checkpoint blockade is feasible in selected patients, but its response kinetics and toxicity profile differ from immunochemotherapy. Histology and PD-L1 assessment therefore remain central to regimen selection. Large Chinese and global phase III programs have broadened the evidence base. ESCORT-1st and its final analysis supported camrelizumab plus chemotherapy, ORIENT-15 established sintilimab plus chemotherapy, and JUPITER-06 demonstrated benefit with toripalimab plus chemotherapy in untreated advanced ESCC (29–32). RATIONALE-306 confirmed a first-line survival advantage with tislelizumab plus chemotherapy, while RATIONALE-302 supported tislelizumab over chemotherapy after progression (33, 34). Patient-reported outcomes from ORIENT-15 further indicate that survival improvement need not require deterioration in global health status, although trial eligibility may underrepresent frail patients encountered in practice (35). Together, ORIENT, ESCORT, JUPITER, and RATIONALE show that domestic PD-1 agents are supported by randomized data and should be integrated into regionally relevant reviews. In localized disease, CheckMate 577 established adjuvant nivolumab after neoadjuvant chemoradiotherapy and R0 resection for patients with residual pathological disease, approximately doubling median disease-free survival compared with placebo (36). This indication differs biologically and clinically from perioperative gastric immunochemotherapy: treatment is selected after chemoradiation, surgery, and pathological risk assessment. The trial therefore supports risk-adapted adjuvant immune therapy rather than indiscriminate preoperative escalation (36). Overall-survival maturation, recurrence patterns, and post-recurrence therapy remain important for defining long-term value.

### 3.3 Colorectal cancer

MSI-high/dMMR colorectal cancer provides the clearest example of biomarker-defined immunotherapy. KEYNOTE-177 established pembrolizumab as first-line therapy for metastatic MSI-high/dMMR colorectal cancer by prolonging progression-free survival and producing durable responses (37). CheckMate 8HW subsequently showed that nivolumab plus ipilimumab provides superior progression-free survival compared with chemotherapy and, in a separate comparison, with nivolumab alone, supporting dual checkpoint blockade as a potent first-line strategy (38). These results should not be generalized to microsatellite-stable disease, where low effective neoantigenicity, T-cell exclusion, liver-metastasis-associated tolerance, and myeloid suppression continue to limit activity. Neoadjuvant studies suggest that immune sensitivity may be even greater in untreated localized dMMR tumors. NICHE-2 reported major pathological responses in nearly all treated patients with locally advanced dMMR colon cancer after short-course nivolumab plus ipilimumab, while dostarlimab produced sustained clinical complete responses permitting nonoperative management in a small cohort with locally advanced dMMR rectal cancer (39, 40). These results are practice changing in concept but require disciplined surveillance, longer follow-up, organ-specific interpretation, and preservation of salvage options. For microsatellite-stable colorectal cancer, rational trials should enrich for biologically plausible subgroups rather than combine checkpoint inhibitors with targeted agents indiscriminately.

### 3.4 Hepatocellular and biliary tract cancers

Hepatocellular carcinoma (HCC) is shaped by chronic inflammation, cirrhosis, viral infection, vascular abnormalities, and liver-specific immune tolerance. IMbrave150 established atezolizumab plus bevacizumab as a first-line standard, demonstrating that vascular endothelial growth factor blockade can complement PD-L1 inhibition when bleeding risk and portal hypertension are appropriately assessed (41). The HIMALAYA program validated the STRIDE regimen of a priming tremelimumab dose plus durvalumab, with durable survival maintained on extended follow-up (42). These regimens differ in contraindications: anti-VEGF therapy requires attention to varices and bleeding, whereas CTLA-4-containing therapy increases immune-mediated toxicity. Clinical trials have expanded first-line options. ORIENT-32 showed superiority of sintilimab plus a bevacizumab biosimilar over sorafenib, CARES-310 demonstrated longer survival with camrelizumab plus rivoceranib, and RATIONALE-301 established noninferior overall survival with tislelizumab versus sorafenib together with a higher response rate (43–45). Patient-reported outcomes from RATIONALE-301 provide additional evidence that efficacy should be assessed alongside symptoms and functioning (46). LEAP-002 did not meet its prespecified statistical thresholds for pembrolizumab plus lenvatinib versus lenvatinib, reminding the field that mechanistic plausibility and early response rates do not guarantee phase III success (47). In advanced biliary tract cancer, TOPAZ-1 established durvalumab plus gemcitabine-cisplatin, and updated follow-up confirmed a persistent survival advantage (48, 49). KEYNOTE-966 independently showed that pembrolizumab added to gemcitabine-cisplatin prolongs overall survival (50). Absolute gains are modest, and biliary tract cancers remain molecularly diverse; FGFR2, IDH1, HER2, BRAF, MSI/dMMR, and other alterations may direct targeted or immune treatment in selected patients. Future studies should determine sequencing and combinations without obscuring the contribution of each active component.

### 3.5 Pancreatic ductal adenocarcinoma

Unselected PDAC remains resistant to checkpoint inhibitor monotherapy because immune exclusion, poor antigen presentation, stromal barriers, and suppressive myeloid networks coexist. MSI-high/dMMR status identifies a rare responsive subgroup, but prevalence is too low to alter outcomes for most patients (11). The POLO trial has therefore been removed from this immunotherapy discussion: olaparib maintenance is a DNA-repair-directed therapy for germline BRCA-mutated PDAC, not evidence of immune efficacy. Its relevance belongs in precision-targeted therapy or in hypothesis-generating studies of how DNA damage may interact with immunity. More credible immune strategies attempt to generate or deliver antitumor specificity. Personalized RNA neoantigen vaccination after surgery induced *de novo* T-cell responses associated with delayed recurrence in a phase I study, and extended analyses demonstrated long-lived vaccine-expanded CD8-positive T-cell clones (51, 52). These observations are biologically persuasive but require randomized confirmation and scalable manufacturing. Oncolytic reovirus pelareorep combined with pembrolizumab and chemotherapy showed immune activation and disease control in a small phase Ib cohort, while intratumoral talimogene laherparepvec was feasible in a phase I pancreatic study but did not establish efficacy (53, 54). For PDAC, immune conversion should be demonstrated through paired tissue, clonal T-cell tracking, and survival endpoints rather than inferred from peripheral cytokines alone.

## 4 Challenges and emerging strategies in immunotherapy for gastrointestinal malignancies

### 4.1 Mechanisms of immunotherapy resistance and potential strategies

Primary resistance may arise before therapy through insufficient immunogenicity, HLA loss, defective antigen processing, T-cell exclusion, suppressive myeloid cells, and fibroblast-derived TGF-β. Acquired resistance emerges when checkpoint blockade selects antigen-loss variants, interferon-insensitive clones, alternative inhibitory receptors, or spatially protected tumor niches (4, 8). A pan-cancer T-cell atlas identified stress-associated states linked to checkpoint resistance, illustrating that conventional exhaustion markers do not fully capture functional competence (7). Mechanistic studies should distinguish correlation from dependency by combining spatial profiling, perturbation, and longitudinal sampling. Resistance also varies by metastatic site and treatment history. Liver metastases can systemically constrain antitumor immunity, while chemotherapy, radiation, antiangiogenic therapy, and antibiotics alter the cellular and microbial context in which checkpoint blockade operates. Single-cell studies in colon cancer show that myeloid-targeted interventions can remodel distinct suppressive populations rather than uniformly deplete macrophages (6). The appropriate response is not maximal combination breadth but selection of the dominant, measurable resistance program.

### 4.2 Cellular therapies, bispecific antibodies, and oncolytic viruses

CLDN18.2 has moved cellular therapy in gastrointestinal cancer from anecdote to randomized evidence. Phase I testing of satricabtagene autoleucel (satri-cel; CT041) showed antitumor activity in heavily pretreated CLDN18.2-positive gastrointestinal cancers, with cytokine-release syndrome and hematologic toxicity generally manageable in specialized centers (55). In the randomized phase II CT041-ST-01 trial, satri-cel improved progression-free survival versus physician’s choice in previously treated advanced gastric or GEJ cancer (56). China subsequently approved satri-cel for CLDN18.2-positive, HER2-negative advanced gastric or GEJ adenocarcinoma, representing the first approved CAR-T product for a solid tumor (57). This advance is clinically substantive, but antigen heterogeneity, trafficking, lymphodepletion, cytopenias, cytokine-release syndrome, manufacturing time, and cost remain limiting. TCR-T expand the target space to intracellular antigens presented by HLA molecules. NY-ESO-1-directed TCR-T produced responses and cytokine-release syndrome in a first-in-human solid-tumor study, whereas individualized TCR gene therapy targeting mutant KRAS generated objective regression in pancreatic cancer (58, 59). Earlier adoptive transfer of KRAS G12D-reactive T cells produced regression in metastatic colorectal cancer but also documented immune escape through loss of the restricting HLA allele (60). TCR-T therefore offers precision unavailable to surface-directed CAR-T, but HLA restriction, antigen loss, tumor heterogeneity, and complex manufacturing narrow eligibility. Bispecific antibodies provide an off-the-shelf alternative. Preclinical studies established CLDN18.2 as a tractable target for CD3-engaging and antibody-drug-conjugate formats, and CLDN18.2-dependent 4-1BB agonism was designed to localize costimulation while limiting systemic toxicity (61, 62). In phase I testing, the CLDN18.2xCD3 antibody IBI389 produced preliminary activity in gastric, GEJ, pancreatic, and other digestive cancers, with cytokine-release syndrome requiring active management (63). Cadonilimab has already supplied phase III evidence for a checkpoint-bispecific strategy in gastric cancer (22). These modalities should be discussed by mechanism and maturity rather than grouped as uniformly established. Oncolytic viruses can lyse tumor cells, release antigens, induce innate sensing, and create a T-cell-inflamed microenvironment, but delivery and antiviral immunity complicate translation. Pelareorep combined with chemotherapy and pembrolizumab produced pharmacodynamic immune activation in PDAC, whereas intratumoral talimogene laherparepvec established feasibility without proving survival benefit (53, 54).

### 4.3 Combination therapeutic strategies

Combination regimens superimpose rather than merely add toxicities. Checkpoint blockade contributes dermatitis, colitis, hepatitis, pneumonitis, endocrinopathies, and less common neurologic or cardiac events; chemotherapy adds cytopenias, infection, neuropathy, nausea, and mucosal injury; antiangiogenic agents increase hypertension, proteinuria, thrombosis, bleeding, and impaired wound healing (64). In HCC, baseline cirrhosis can obscure immune hepatitis, while bevacizumab-containing therapy requires endoscopic assessment and management of variceal risk. CAR-T and CD3 bispecific antibodies add cytokine-release syndrome, cytopenias, infection, and occasionally neurotoxicity (63, 64). Toxicity attribution becomes difficult when diarrhea, transaminase elevation, fatigue, or cytopenia has several plausible causes. Management should therefore define baseline organ function, anticipated overlapping events, dose-hold rules, corticosteroid thresholds, infection prophylaxis, and multidisciplinary escalation before treatment starts (64). Real-world pharmacovigilance is especially important because pivotal trials exclude many patients with autoimmune disease, organ transplantation, poor performance status, or advanced hepatic dysfunction. A systematic analysis of checkpoint-inhibitor toxicity underscores that organ-specific risk depends on agent, combination, exposure, and patient context rather than a single class-wide incidence (65).

### 4.4 Biomarker development and personalized immunotherapy

Static biomarkers capture only one time point in an evolving immune response. PD-L1, MSI/dMMR, TMB, Epstein-Barr virus status, HLA genotype, tumor-infiltrating lymphocytes, and anatomical disease distribution should be integrated with regimen and stage. Plasma circulating tumor DNA dynamics correlated with the efficacy of PD-1 inhibitor plus chemotherapy in advanced gastric cancer and may provide earlier response information than imaging, although clearance is not equivalent to eradication of viable disease (66). Prospective trials must define collection timing, assay sensitivity, and decision rules before ctDNA can direct treatment escalation or de-escalation. The gut microbiome is another potential modifier but remains vulnerable to geography, diet, antibiotics, sequencing methods, and reverse causality. In advanced HCC, baseline microbial composition was associated with nivolumab response, providing a rationale for validation rather than immediate microbiome-directed treatment (67). Multi-omics models can combine tumor genomics, spatial immune states, blood biomarkers, microbiota, imaging, and clinical variables, but high dimensionality increases overfitting and reduces transportability. Useful biomarkers must be analytically reproducible, prospectively locked, externally validated, and shown to improve a treatment decision rather than merely correlate with outcome.

## 5 Conclusion

Immunotherapy has become a disease- and biomarker-specific component of gastrointestinal oncology rather than a uniform treatment class. Randomized trials support checkpoint blockade in advanced gastric, esophageal, colorectal, hepatocellular, and biliary tract cancers, while perioperative evidence now differentiates successful strategies such as MATTERHORN and CheckMate 577 from less definitive programs such as KEYNOTE-585. Chinese-developed antibodies have generated mature phase III evidence and should be integrated into global treatment frameworks. In contrast, pancreatic cancer and microsatellite-stable colorectal cancer still require biologically grounded approaches that create, deliver, or sustain tumor-reactive immunity.

The next phase will be shaped by CLDN18.2-directed CAR-T cells, TCR-engineered cells, bispecific antibodies, personalized vaccines, and oncolytic viruses, but each platform must be judged by randomized efficacy, manufacturability, biomarker reproducibility, and toxicity rather than novelty. Combination therapy should target a demonstrated resistance mechanism and include prospective plans for overlapping adverse events. Longitudinal tissue and liquid biopsies may connect immune-state change with clinical outcome, provided assays are standardized and externally validated. The field should prioritize trials that distinguish prognostic from predictive biomarkers, incorporate patient-reported outcomes, and test rational sequencing. Equitable implementation will also require regional evidence, affordable diagnostics, scalable manufacturing, and health systems capable of managing toxicity. Progress will depend on selecting the right immune intervention for the right tumor ecosystem and disease stage while preserving quality of life and access.
